# Intraclonal Genome Stability of the Metallo-β-lactamase SPM-1-producing *Pseudomonas aeruginosa* ST277, an Endemic Clone Disseminated in Brazilian Hospitals

**DOI:** 10.3389/fmicb.2016.01946

**Published:** 2016-12-05

**Authors:** Ana P. B. Nascimento, Mauro F. Ortiz, Willames M. B. S. Martins, Guilherme L. Morais, Lorena C. C. Fehlberg, Luiz G. P. Almeida, Luciane P. Ciapina, Ana C. Gales, Ana T. R. Vasconcelos

**Affiliations:** ^1^Laboratório de Bioinformática, Laboratório Nacional de Computação CientíficaPetrópolis, Brazil; ^2^Laboratório Alerta, Division of Infectious Diseases, Department of Internal Medicine, Escola Paulista de Medicina, Universidade Federal de São PauloSão Paulo, Brazil

**Keywords:** drug resistance, comparative genomics, pathogenic bacteria, antimicrobial resistance, carbapenemase, Gram-negative bacilli

## Abstract

Carbapenems represent the mainstay therapy for the treatment of serious *P. aeruginosa* infections. However, the emergence of carbapenem resistance has jeopardized the clinical use of this important class of compounds. The production of SPM-1 metallo-β-lactamase has been the most common mechanism of carbapenem resistance identified in *P. aeruginosa* isolated from Brazilian medical centers. Interestingly, a single SPM-1-producing *P. aeruginosa* clone belonging to the ST277 has been widely spread within the Brazilian territory. In the current study, we performed a next-generation sequencing of six SPM-1-producing *P. aeruginosa* ST277 isolates. The core genome contains 5899 coding genes relative to the reference strain *P. aeruginos*a PAO1. A total of 26 genomic islands were detected in these isolates. We identified remarkable elements inside these genomic islands, such as copies of the *bla*_SPM−1_ gene conferring resistance to carbapenems and a type I-C CRISPR-Cas system, which is involved in protection of the chromosome against foreign DNA. In addition, we identified single nucleotide polymorphisms causing amino acid changes in antimicrobial resistance and virulence-related genes. Together, these factors could contribute to the marked resistance and persistence of the SPM-1-producing *P. aeruginosa* ST277 clone. A comparison of the SPM-1-producing *P. aeruginosa* ST277 genomes showed that their core genome has a high level nucleotide similarity and synteny conservation. The variability observed was mainly due to acquisition of genomic islands carrying several antibiotic resistance genes.

## Introduction

*Pseudomonas aeruginosa* is a ubiquitous microorganism present in many diverse ecological niches, including water, soil, plants, animals, and humans. The ability of *P. aeruginosa* to survive on minimal nutritional requirements and to tolerate a variety of physical conditions has allowed this organism to persist in environmental and hospital settings (Pier and Ramphal, 2010). Carbapenems represent the main therapy for the treatment of serious *P. aeruginosa* infections. However, the emergence of carbapenem resistance has jeopardized the clinical use of this important class of compounds (Papp-Wallace et al., 2011). Among *P. aeruginosa*, hyperproduction of AmpC and/or metallo-β-lactamases coupled with alteration in the outer membrane permeability represent the main mechanism of carbapenem resistance (Lister et al., 2009; Papp-Wallace et al., 2011). In Brazil, *P. aeruginosa* is an important pathogen in the nosocomial environment. According to the latest report of the Brazilian Health Surveillance Agency^1^, *P. aeruginosa* ranked as the fifth most common pathogen causing catheter-related bloodstream infections in adult patients hospitalized at Brazilian intensive care units. Among the 2480 *P. aeruginosa* reported, nearly 42% were resistant to carbapenems. To date, the production of SPM-1, São Paulo metallo-β-lactamase, has been the most common mechanism of carbapenem resistance identified in *P. aeruginosa* isolated from Brazilian medical centers (Toleman et al., 2002; Scheffer et al., 2010; Rossi, 2011). However, unlike other carbapenemases such as NDM, IMP, and KPC, SPM-1 has been only reported in *P. aeruginosa* isolates. Previous studies have shown the presence of a SPM-1-producing *P. aeruginosa* clone belonging to the ST277, clone SP, widely spread within the Brazilian territory (Gales et al., 2003; Scheffer et al., 2010; Silva et al., 2011; Silveira et al., 2014).

This study was undertaken to determine the possible presence of genetic factors associated with the resistance and persistence of this clone within Brazilian institutions In addition, we aimed to compare the genome of the SPM-1-producing *P. aeruginosa* isolates collected from a single intensive care unit over a 9-year period to evaluate whether this clone had suffered any temporal changes compared with the index isolate.This is the first study to date to comprehensively evaluate and compare the complete genome of the SPM-1-producing *P. aeruginosa* ST277 isolates, as the genome of few SPM-1-producing *P. aeruginosa* strains have only been partially analyzed (Boyle et al., 2012; Silveira et al., 2014; van Belkum et al., 2015).

## Methods

### Bacterial strains, culture conditions, and DNA isolation

We studied six SPM-1-producing *P. aeruginosa* isolates, including the index isolate PA1088 (previously named 48-1997A), which was the first reported clinical isolate to carry *bla*_SPM−1_ (Toleman et al., 2002). The remaining five isolates were recovered from distinct patients admitted to a single intensive care unit between the years 2003 and 2012 (Table 1). All isolates were collected from the same tertiary teaching hospital located in the city of São Paulo, Brazil. The presence of *bla*_SPM−1_ was initially confirmed by PCR and DNA sequencing (BigDye Terminator Cycle Sequencing, Applied Biosystems, Foster City, USA) using primers previously described (Mendes et al., 2004). For whole genome sequencing, the bacteria were grown overnight in LB broth (Oxoid, Basingstoke, England) at 37°C. Total DNA was extracted using the Qiamp DNA Stool Kit (Qiagen, Hilden, Germany) according to the manufacturer's instructions. The DNA concentration was measured in a NanoVue digital spectrophotometer (GE Healthcare Life Sciences, New Jersey, USA) and submitted to the Unidade de Genômica Computacional Darcy Fontoura de Almeida (UGCDFA) of Laboratório Nacional de Computação Científica (LNCC) for further analysis.

**Table 1:** **Bacterial isolates sequenced in this work**.

**ID isolate**	**Year of isolation**	**Clinical specimens**	**PFGE**
PA1088	1997	Urine	A
PA3448	2003	Bloodstream	A2
PA7790	2006	Tracheal aspirate	A1
PA8281	2007	Tracheal aspirate	A1
PA11803	2011	Bloodstream	A3
PA12117	2012	Bloodstream	A2

### DNA sequencing, genome assembly, genome annotation, and comparative genomics

Six whole genome sequencing libraries were generated using the Illumina TruSeq DNA PCR-free sample preparation kit with a median insert size of 550 bp according to the manufacturer's protocols. Briefly, 2 μg of genomic DNA was sheared using a Covaris M220 Focused-ultrasonicator, end-repaired, A-tailed, and adapter ligated. Library quantification was carried out by real-time PCR. Libraries were pooled together in equimolar amounts and sequenced by an Illumina MiSeq instrument in one 2 × 300 bp paired-end run. Genome assembly was performed using Newbler version 3.0. In addition, Celera assembler version 8.2 was used to close eventual gaps. Gaps within scaffolds resulting from repetitive sequences were resolved by *in silico* gap filling. We achieved mean sequence coverage of 170-fold for each of the six genomes. Mauve-based alignment of contigs revealed extensive synteny between the genomes of the six isolates and the reference genome of *P. aeruginosa* PAO1. However, two contigs of 49 kb did not align with chromosomal sequences. Notably, the mean sequence coverage for these putative extrachromosomal contigs was 3-fold higher than that observed for the syntenic contigs. Moreover, the two contigs showed different start points in different assemblies, indicating a circular sequence. The System for Automated Bacterial Integrated Annotation (SABIA) pipeline was used for gene prediction and automatic annotation followed by manual validation (Almeida et al., 2004). After annotation, the genomes were analyzed by the Bidirectional Best-Hits (BBH) clustering method (Overbeek et al., 1999), which compares different genomes with each other using the BLAST program (Altschul et al., 1997) to identify pairs of corresponding genes (clusters) and to recognize the best hit in other genomes. The parameters applied were 90% coverage, 90% of similarity and an *e* < 10^−5^. The GView Server (Petkau et al., 2010) was used to obtain the sequence of pan, core and unique genome of the six isolates using the *P. aeruginosa* PAO1 strain as a reference when necessary, with a minimum identity of 90% and an *e* < 10^−5^. The genomes of the six SPM-1-producing *P. aeruginosa* isolates were deposited in Genbank repository under the accession numbers: CP015001 (PA1088); LVWC01000000 (PA3448 contigs and plasmid); CP014999 (PA7790); CP015000 (PA7790 plasmid); CP015002 (PA8281); CP015003 (PA11803) and LVXB01000000 (PA12117).

### Phylogenetic analysis and multilocus sequence typing

We used all conserved open reading frames (ORFs) among our six strains, the reference genome PAO1 (Stover et al., 2000), 11 ST277 strains with genome available:19BR (GCA_000223945.2), 213BR (GCA_000223965.2), 9BR (GCA_000223925.2), BWHPSA041 (GCA_000520375.1), AZPAE12409 (GCA_000797005.1), AZPAE14819 (GCA_000795205.1), AZPAE14821 (GCA_000795235.1), AZPAE14822 (GCA_000795265.1), AZPAE14853 (GCA_000789905.1), AZPAE14923 (GCA_000791205.1), CCBH4851 (GCA_000763245.1) and a single MLST locus variant strain: BWHPSA007 (GCA_000481565.1) to reconstruct the phylogeny. A total of 5 042 protein sequences were concatenated applying neighbor joining, minimum evolution, UPGMA and maximum parsimony methods for reconstruct initial trees using Poisson method, uniform rates among sites, complete deletion treatment to gaps and bootstrap 100 with MEGA software (Kumar et al., 2016). The tree was visualized using the TreeView tool (Page, 1996).

The seven housekeeping genes *acs, aro, gua, mut, nuo, pps*, and *trp* were selected according to the multilocus sequence typing (MLST) scheme for *P. aeruginosa*^2^ to confirm the allelic profiles of the six isolates.

### Genomic islands and insertion sequences identification

Genomic islands (GIs) are segments of DNA mostly acquired by horizontal gene transfer. IslandViewer 3 (Dhillon et al., 2015) and PIPS (Soares et al., 2012) software were applied to detect genomic islands. IslandViewer 3 is based on sequence composition and comparative genomic methods, which may result in a prediction of false positives GIs, whereas PIPS includes the detection of virulence factors, hypothetical proteins, and flanking tRNAs in its analysis, which may exclude regions that do not meet these parameters. Both outputs were validated manually by observing the following criteria: (i) atypical G+C content; (ii) presence of mobile elements; (iii) adjacency to tRNA genes; (iv) size above 5 000 bp; and (v) comparison of the boundaries to the genomic context of the PAO1 reference strain. All criteria should be fulfilled, except for items (ii) and (iii), which were not mandatory to characterize a genomic island. We also used the IS Finder database and tools (Siguier et al., 2006) to identify the insertion sequences (ISs) from *P. aeruginosa* genomes. We considered full elements or fragments with *e* < 10^−6^ in BLASTn searches (Altschul et al., 1997). We also incorporated ORF regions sharing similarities with transposases into genome annotation with the SABIA platform (Almeida et al., 2004).

### Single nucleotide polymorphisms analysis

To identify possible polymorphisms in the six *P. aeruginosa* samples, we performed a single nucleotide polymorphism (SNP) calling using the PAO1 genome (NC_002516) as a reference. Briefly, the FASTA genome and GTF gene coordinates files were retrieved from NCBI. The deep sequencing libraries files were quality checked with the FastQC tool^3^ and trimmed with the FASTX_Toolkit^4^. The trimmed reads from the six samples were mapped separately against the PAO1 genome with the Bowtie 2 (Langmead and Salzberg, 2012) mapper with one mismatch per seed region (20 nucleotides in length), using three different seed regions for each read with repetitive regions and trying to extend 20 nucleotide after mapped seed region (very sensitive preset). The resulting mapping files were treated with the SAMtools program (Li et al., 2009); only mapping reads with a map quality (mapQ) greater than 30 were kept. The Picard mark duplicates tool^5^ was used to flag putative sequencing artifacts, such as optical duplicates. The Genome Analysis Toolkit (GATK) (McKenna et al., 2010) was used to call the variants using default parameters. The SNPs were annotated with the SnpEff tool (Cingolani et al., 2012) and custom Python scripts. Only SNPs with coverage larger than 10 reads were considered in further analyses. To find SNPs among the six *P. aeruginosa* isolates, the PA1088 genome was used as a reference, and the SNP call was performed as previously mentioned. In addition to the SNP call analysis, we performed a BLASTp search (Altschul et al., 1997), using the protein sequences of the six isolates against the PAO1 encoded proteins. This approach enabled us to find any polymorphism that was not detected by the previous method.

### Susceptibility testing, pulsed-field gel electrophoresis, and qRT-PCR

Antimicrobial susceptibility testing was performed by broth microdilution, and the results were interpreted according to the criteria of the European Committee on Antimicrobial Susceptibility Testing^6^. *P. aeruginosa* ATCC 29853 and *Escherichia coli* ATCC 25922 were tested as quality control strains. The genetic relatedness was initially determined by pulsed-field gel electrophoresis (PFGE) using SpeI enzyme (200 V [6 V/cm]; 13°C; switch time initial 5.0 and final, 60.0; 23 h) (Pfaller et al., 1992), and the results (Figure S1) were interpreted as previously recommended (Tenover et al., 1995). Two copies of *bla*_SPM−1_ were identified in the isolates PA3448 and PA8281. To confirm whether the *bla*_SPM−1_ multiple copies had led to an increase in transcriptional levels, qRT-PCR experiments were carried out. Total RNA was collected from SPM-1-producing *P. aeruginosa* isolates using the RNeasy Mini Kit (Qiagen, Hilden, Germany) with addition of RNase-free DNase (Qiagen, Hilden, Germany). Reverse transcription of the extracted RNA was performed using the High Capacity cDNA Reverse Transcription Kit (Life Technologies, Carlsbad, CA, USA). The pair of primers used for the amplification of the *bla*_SPM−1_ and 16S rDNA genes were as previously reported (Mendes et al., 2007). Transcripts were quantified in triplicate using SYBR® Green PCR Master Mix (Life Technologies, Carlsbad, CA, USA) and the 7500 Real Time system (Life Technologies, Carlsbad, CA, USA). The 16S rDNA gene was used as a reference to normalize the relative amount of mRNA. The *bla*_SPM−1_ transcriptional levels were compared using PA1088 as the reference strain because it has been known to carry a single copy of *bla*_SPM−1_. The transcriptional level of genes encoding efflux pumps (*mexB, mexD, mexF*, and *mexY)*, and the OprD porin (*oprD*) was also studied but using the PAO1 as the reference strain. Mean values (± standard deviations) of mRNA levels obtained in triplicate were calculated. Strains showing mRNA values of >5-fold for *mexD, mexF*, or *mexY* or >2-fold for *mexB* were considered to overexpress these genes (Cabot et al., 2011).

## Results

### General genomic features

A summary of the genomic features of the six newly sequenced genomes of *P. aeruginosa* isolates is provided in Table 2. The whole genome size ranged from 6,643,783 to 7,018,690 bp, with only the PA3448 and PA7790 isolates observed to carry a plasmid. Although the isolates had similar G+C contents, it was possible to note some differences in the total number of coding sequences (CDSs) according to the variation in the chromosome size. An overview of the whole genome homology among the *P. aeruginosa* isolates, core genome and unique regions is presented in Figure 1.

**Table 2:** **General features of the genomes of SPM-1-producing ***P. aeruginosa*** ST277 clinical isolates relative to the reference strain ***P. aeruginosa*** PAO1**.

**Feature**	**Isolate**						
	**PA1088**	**PA3448**	**PA7790**	**PA8281**	**PA11803**	**PA12117**	**PAO1**
Chromosome size (bp)	6,721,480	6,794,242	7,018,690	6,928,736	7,006,578	6,643,782	6,264,404
Plasmid size (bp)	–	49,094	49,021	–	–	–	–
G+C content (%)	66.14	66.12	65.96	66	65.97	66.22	66.55
Total no. CDSs	6199	6274	6540	6426	6582	6115	5571
Average CDSs length (bp)	962.59	956.18	945.97	954.73	943.86	964.17	1 002.45
Known proteins	5160	5199	5309	5273	5268	5109	3286
Hypothetical proteins	1012	1039	1198	1117	1281	972	2270
No. of rRNAs	12	12	12	12	12	12	13
No. of tRNAs	64	64	67	64	67	64	63

**Figure 1:**

**Circular map depicting the unique regions of each SPM-1-producing ***P. aeruginosa*** isolate relative to the reference strain PAO1**. The red ring represents the core genome shared by all isolates. The outermost, interspaced ring represents the localization of the predicted genomic islands found in each isolate.

A functional classification based on KEGG analysis assigned the CDSs into the 19 main categories was performed (Table S1). All isolates showed a conserved distribution of CDSs among the categories relative to the reference strain PAO1; one exception was the replication and repair category, in which 10 additional CDS were found (Table S2), such as the subunits A and B of excinuclease ABC, a single-stranded DNA-binding (SSB) protein and a DNA methyltransferase present in all six isolates. Interestingly, these additional CDSs involved in replication and repair were located in the genomic islands found among the six isolates.

#### Plasmids

The isolates PA3448 and PA7790 carried a plasmid with a size of approximately 49 Kb and a G+C content of 58.8%. A major portion of this plasmid (89%) shared 96% of its identity with a chromosomal region of *P. aeruginosa* PSE305 (Wright et al., 2015). The majority of ORFs were predicted to encode hypothetical proteins, except for those encoding proteins that could be related to the type II secretion system (T2SS) (1 ORF), plasmid stabilization and mobilization (4 ORFs), DNA replication and repair (3 ORFs), and other functions assigned by homology Table S3). The plasmids were identical except for one CDS present only in PA3448's plasmid, ORF 44, which encodes a putative adhesin (Figure S2).

### Phylogenetic analysis and allelic profiles

**Figure 2:**
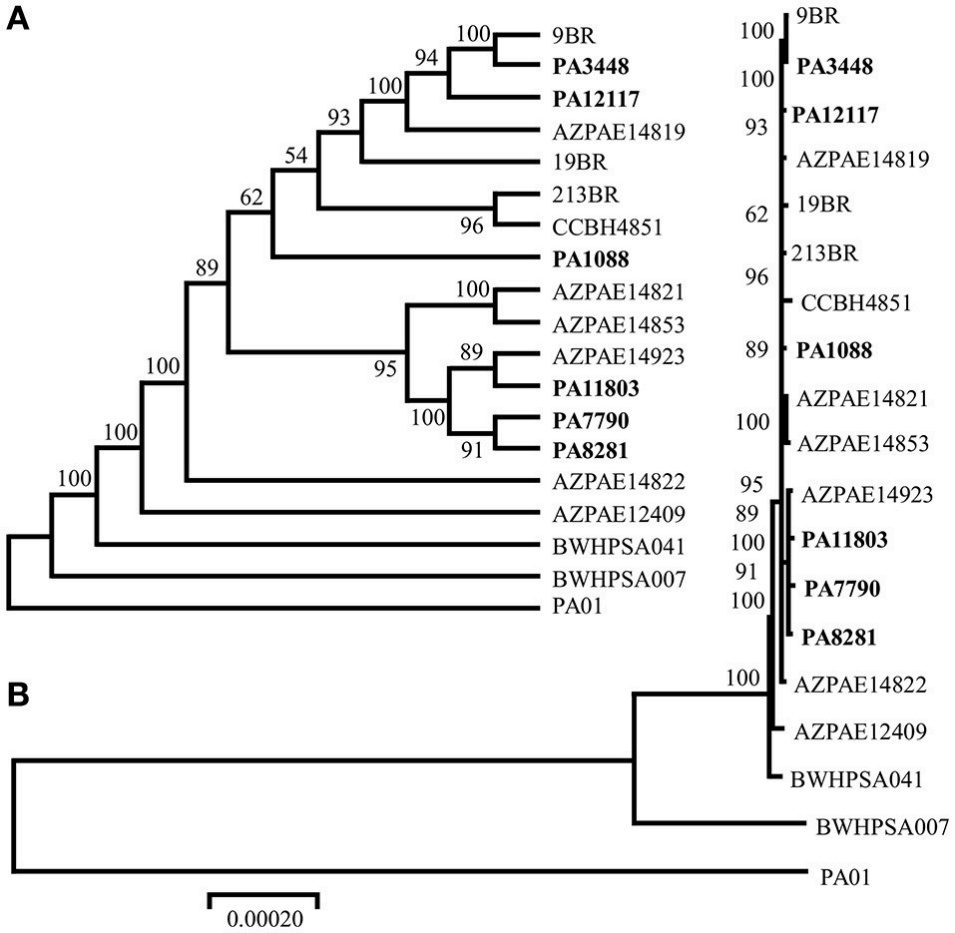
**Phylogenic tree of the six sequenced ***P. aeruginosa*** isolates, the reference genome PAO1, the 11 ST277 strains: 19BR (GCA_000223945.2), 213BR (GCA_000223965.2), 9BR (GCA_000223925.2), BWHPSA041 (GCA_000520375.1), AZPAE12409 (GCA_000797005.1), AZPAE14819 (GCA_000795205.1), AZPAE14821 (GCA_000795235.1), AZPAE14822 (GCA_000795265.1), AZPAE14853 (GCA_000789905.1), AZPAE14923 (GCA_000791205.1), CCBH4851 (GCA_000763245.1), and a single MLST locus variant strain: BWHPSA007 (GCA_000481565.1)**. The numbers indicate the bootstrap value associated with the nodes. **(A)** Consensus tree with neighbor joining method and **(B)** best tree drawing on scale.

The MLST allelic profiles confirmed the expected relationship showing that all isolates were grouped under the same sequence type, ST277. The concatenated ORFs were used to build a phylogenetic tree representing approximately 70% of the size of each genome. Our phylogenic reconstruction showed that the six *P. aeruginosa* isolates formed a monophyletic group with other ST277 strains, which supports the high similarity observed among these isolates (Figure 2). All methods used showed the same result, corroborating the validity of the groups. Among the ST277 strains were found two main groups, one including the PA3448 and PA12117 isolates, and another with PA11803, PA7790, and PA8281 isolates. Only the PA1088 isolate showed an unclear relationship due to its low branch support.

### Comparative genomics

**Figure 3:**
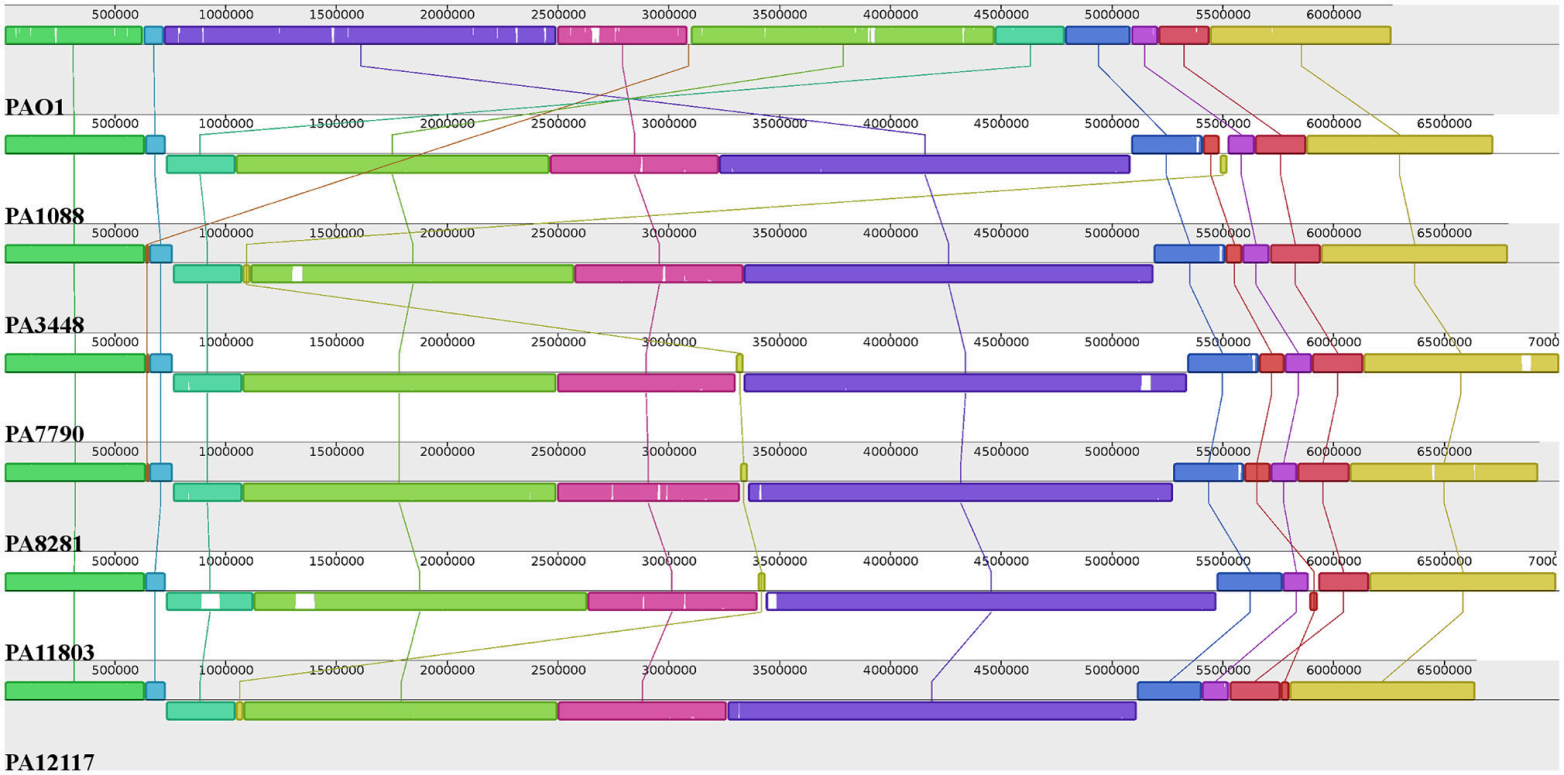
**Pairwise alignment between the ***P. aeruginosa*** chromosomes**. Colors indicate conserved and highly related genomic regions, and white areas identify unique or low-identity regions. Blocks shifted below the horizontal axis indicate segments that align in the reverse orientation relative to the reference strain PAO1.

The overall chromosome organization of the *P. aeruginosa* isolates was compared with that of the reference strain PAO1 using Mauve software (Darling et al., 2004). This analysis revealed a conserved structure among the chromosomes of the SPM-1-producing *P. aeruginosa* isolates. The multiple alignments showed the existence of 11 conserved blocks; however, it was observed that unique regions were also present (Figure 3). Compared with the PAO1 strain, a major rearrangement was observed in all six *P. aeruginosa* clones. This rearrangement is an inversion that could be a result of a homologous recombination between genes encoding a 23S ribosomal RNA (PA0668.4 and PA4280.2 relative to PAO1 strain), which was orientated in opposite directions, and share 99% identity.

#### Comparison of genetic repertoire with *P. aeruginosa* PAO1

The genomes of the six SPM-1-producing *P. aeruginosa* were compared with the genome of the reference strain PAO1 using the BBH method to evaluate the absence or partial homology of CDSs. All six isolates lacked 102 PAO1 ORFs encoding pyocins, phage elements, regulators, transporters, several hypothetical proteins and others. Among these pyocins, two, S2 and S4, were completely absent. In addition, 18 PAO1 ORFs shared only partial homology with predicted ORFs of SPM-1 isolates, including two porins encoded by PA0958 and PA2213 loci, and the transcriptional regulator encoded by PA2020 (Table S4). All six SPM-1-producing *P. aeruginosa* isolates showed a 2 bp deletion of 380 and 381 nucleotides in PA0958 (*oprD*), changing the reading frame and causing a gain of a premature stop codon. The porin encoded by the PA2213 gene also gained a premature stop codon because of a nucleotide change in position 193. The sequence of PA2020 (*mexZ*) lost 19 bp in all isolates, leading to a gain of a premature stop codon.

#### Comparison of the unique and shared genes among the SPM-1-producing *P. aeruginosa* isolates

A core genome containing 5899 coding genes was identified by the BBH method, representing 89–96% of the total number of CDSs of each clone. Genes conserved among all genomes encode proteins contributing mainly to fundamental housekeeping functions. The set of unique genes encountered for each isolate represents between 0.4 and 5% of the total number of CDSs: 38 for PA1088, 56 for PA3448, 111 for PA7790, 75 for PA8281, 334 for PA11803, and 27 for the PA12117 isolate. The majority of unique genes were annotated as hypotheticals because no function could have been attributed. Other genes were mostly associated with phages, transposases and integrases (Table S5). Among the unique genes, we identified PAO1 partial genes, such as *kinB* (encoding a two component system sensor protein) in the PA1088 isolate, *radC* (DNA repair protein) in the PA8281 isolate and *lasR* (transcriptional regulator) in the PA12117 isolate.

### Genomic islands and insertion sequences

**Figure 4:**
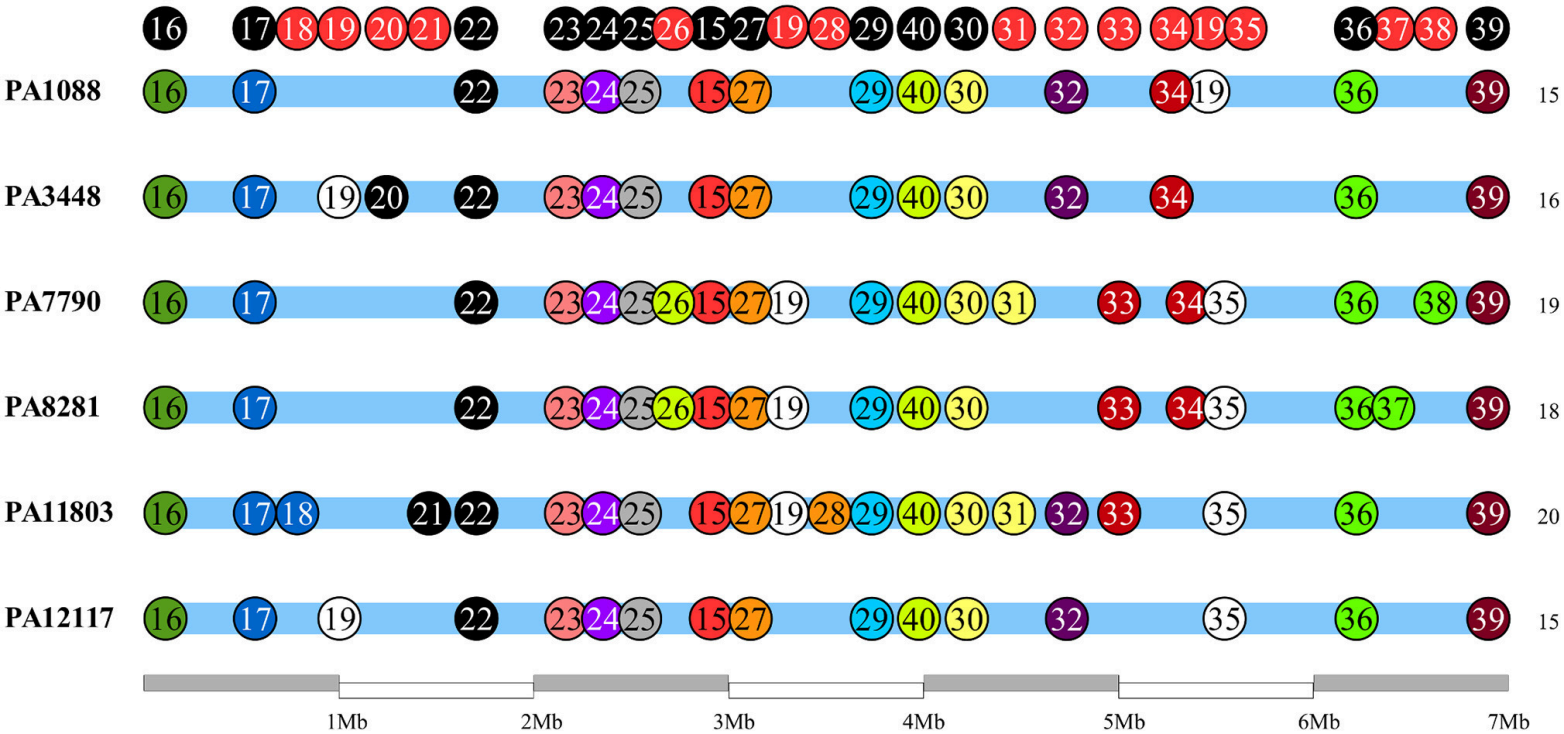
**Schematic overview of genomic island (PAGI) distribution in the six SPM-1 isolates**. Each circle represents a PAGI throughout the bacterial chromosomes. There are a total of 26 PAGIs, and each isolate carries between 16 and 21 PAGI (number at right). The circles at the top represent conserved sites (black) and variable sites (red).

We identified 26 genomic islands in the six studied *P. aeruginosa* isolates. They were named PAGI (*P. aeruginosa* genomic island) and numbered accordingly from 15 to 40, i.e., PAGI-15 to PAGI-40. The size of the smallest region found was 5 914 bp, whereas the largest was 132,631 bp. A total of 14 islands were common to all six isolates, whereas other 6 were unique, 3 of which were present only in PA11803 (Figure 4; Table S6). Most CDSs observed in PAGIs were predicted to encode hypothetical proteins in addition to transposases, integrases and phage-related proteins (Table S7). Genes conferring antibiotic resistance were mainly located in PAGI-15 and -25, such as *bla*_SPM−1_, *bla*_OXA−56_, *rmtD, cmx*, and *sul1*. Genes homologous to those encoding proteins involved in the cell response to a stress condition, such as *hicAB, hipAB*, and *higAB*, were identified in PAGI-17, -24, and -31, respectively. PA1088, PA34448, PA7790, and PA8281 isolates acquired a gene predicted to encode a pyocin, a potent bacteriocin implicated in intraspecific microbial competition, homologous to the S5 type. This gene was carried by PAGI-34. Additionally, PAGI-20, -21, -32, -33, and -38 harbor genes homologous to *prtN, ptrB*, and *prtR*, which are involved in the regulation of pyocin production. Interestingly, PAGI-34 was 63% similar to a previously described island, PAPI-1; this island, in contrast to PAGI-34, did not carry a CRISPR-Cas system. This system, which possesses *cas3, cas5, cas8c, cas7, cas4, cas1*, and *cas2* genes (type I-C), was present in four (PA1088, PA3448, PA7790, PA8281) of the six sequenced isolates, but it was absent in the remaining two isolates. Whereas the CRISPR-Cas system was intact in the PA1088 and PA3448 isolates, it was interrupted by the insertion of another island (PAGI-35) in the PA7790 and PA8281 isolates (Figure S3). PAGI-34 also shared a higher coverage (99%) and similarity (99%) to other mobile elements, the pKLC102-like ICEs, which have been previously reported to carry a type I-C CRISPR-Cas system (van Belkum et al., 2015). PAGI-28, an island present only in PA11803, carried a gene locus predicted to encode proteins homologous to type I-E and type I-F anti-CRISPR systems, namely JBD5-gp34, -gp35, -gp36, and -gp37 phage proteins (Bondy-Denomy et al., 2013; Pawluk et al., 2014).

**Figure 5:**
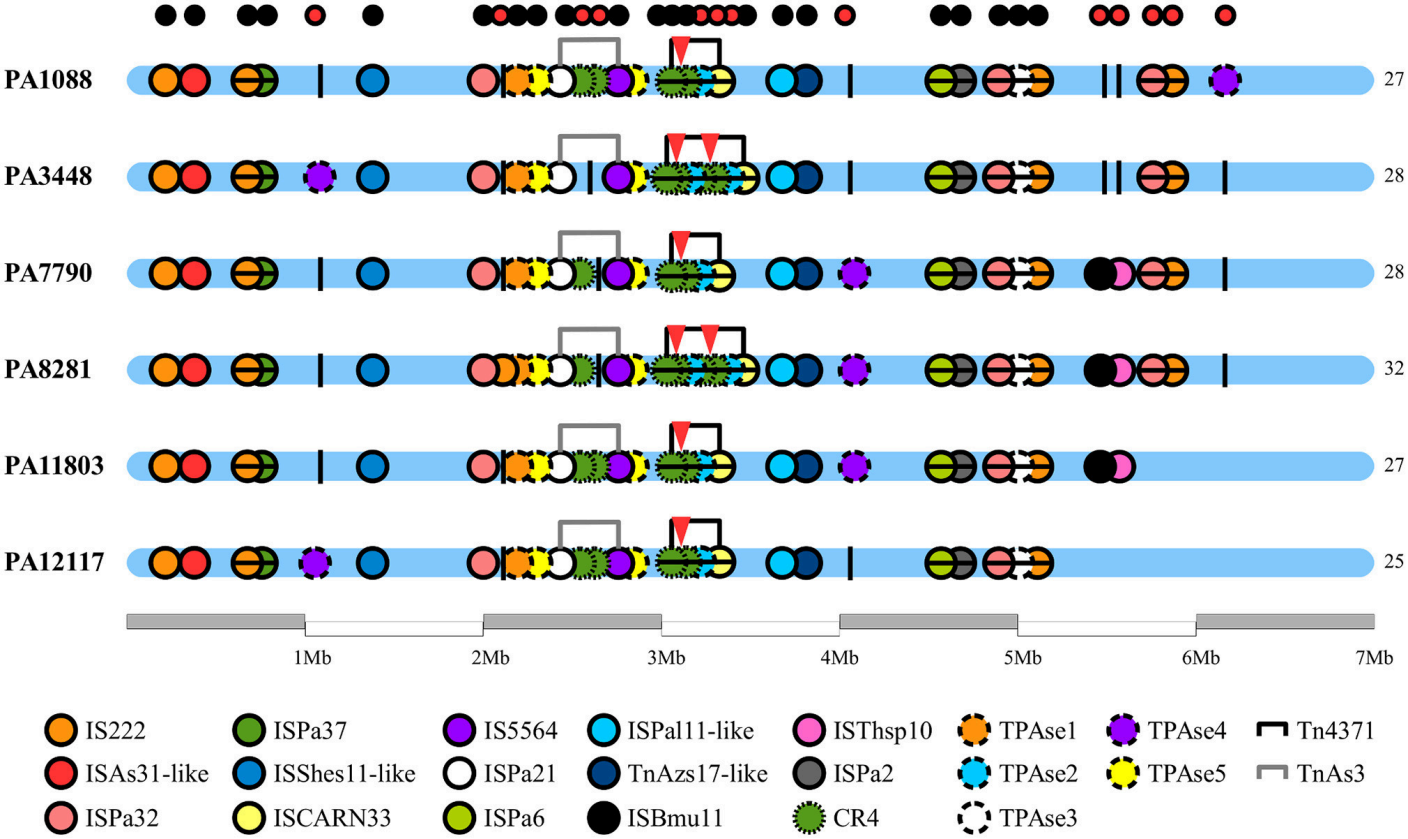
**Chromosome distribution of insertion sequences of ***P. aeruginosa*** genomes**. Each circle represents an insertion sequence (IS) throughout the bacterial chromosome. In the bottom of the figure, the legend differentiates the various ISs by color. The black vertical lines are the empty sites. The small circles at the top of the figure indicate the conservative (black) and variable (red) ISs sites. The red triangles indicate the presence of *spm-1* gene copies.

**Figure 6:**
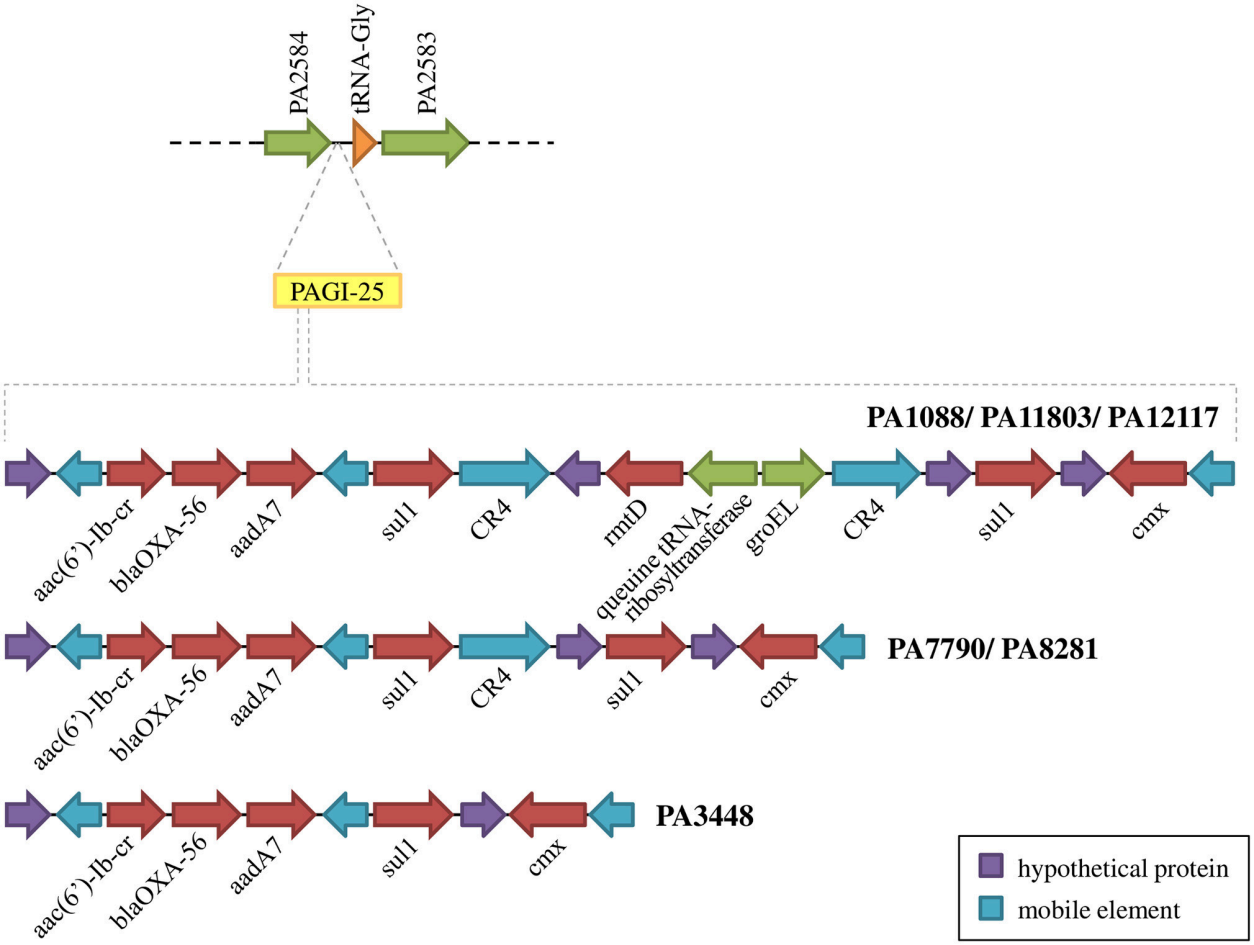
**Schematic overview of PAGI-25 highlighting the region harboring genes conferring acquired antibiotic resistance**.

We identified 20 different types of insertion sequences in the six *P. aeruginosa* isolates; some ISs were found more than one time in the genome, such as IS222, CR4, TPAse5. The overall distribution of ISs was quite similar among the SPM-1 isolates (25–32) (Figure 5). We observed 21 IS sites conserved between all six isolates. Regarding the variable sites, mobile elements such as TPAse4 were observed at different locations in the genome inserted within PAGI-19, which was present in all six isolates at different chromosomal positions (Figure 5; Table S6). Other variable sites comprised the CR4 elements. Two of them were located inside of PAGI-15 but suffered duplication in PA3448 and PA8281 isolates, resulting in an additional copy of the *bla*_SPM−1_ gene. Additional copies of CR4 elements were found in PAGI-25; in PA1088, PA11803, and PA12117, we observed these copies occur next to one copy of the *sul1* gene (absent in PA3448) and another next to the *rmtD* gene (absent in PA3448, PA7790, and PA8281) (Figure 6). The complete list of IS elements identified is presented in Table S8.

### SNPs analysis

To find SNPs in the six *P. aeruginosa* isolates, the complete genome of *P. aeruginosa* PAO1 was used as a reference to perform an SNP call. The same strategy was used to find SNPs among the six isolates using the PA1088 genome as reference.

**Figure 7:**
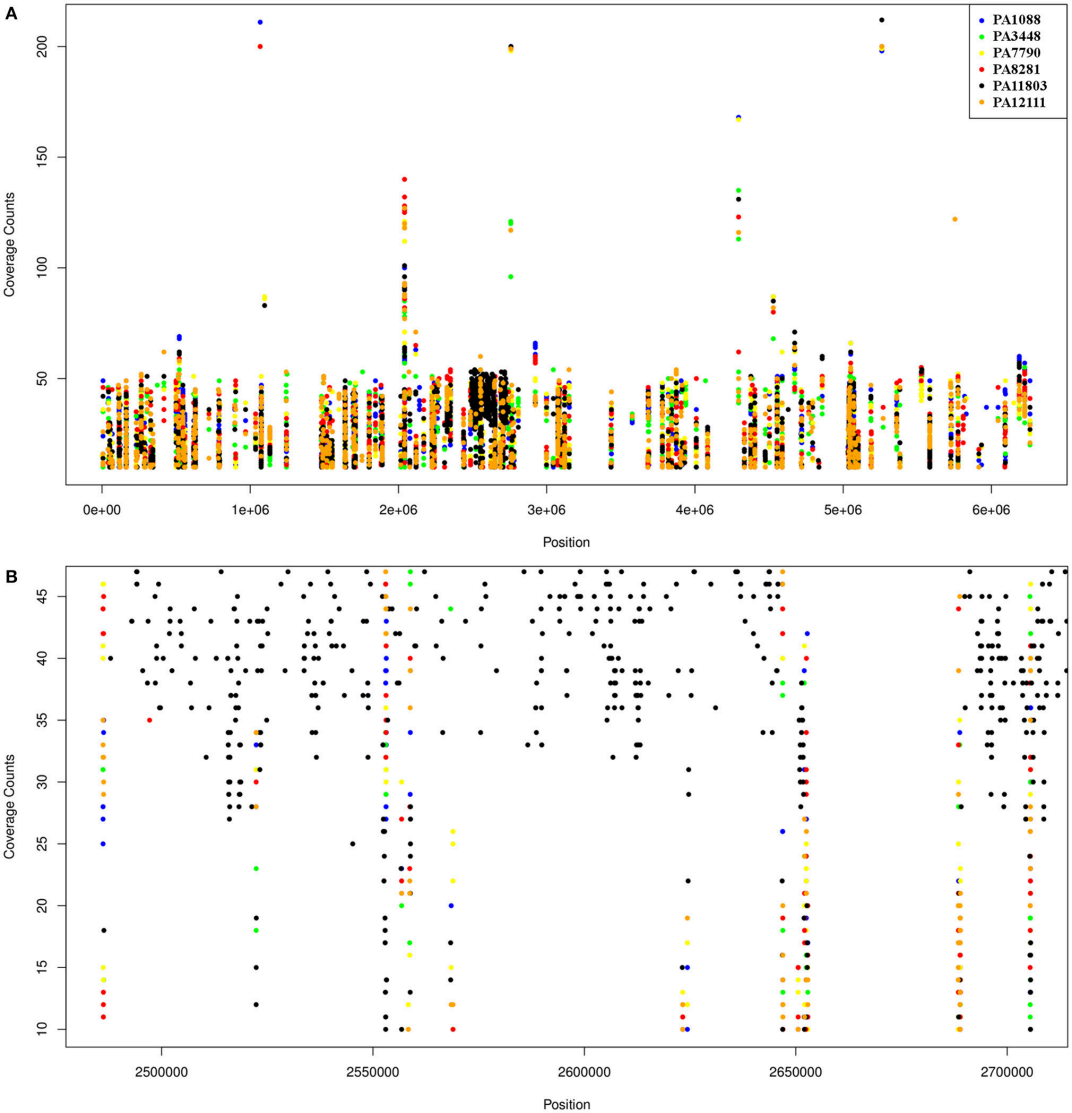
**Schematic representation of SNPs distribution in the six ***P. aeruginosa*** isolates using PAO1 as a reference genome**. Each dot represents an SNP. The coverage counts show how many mapped reads support the SNP. **(A)** SNPs distribution of the six isolates relative to PAO1; the black spot refers to the SNP enriched region in PA11803. **(B)** A higher resolution representation of the PA11803 SNP enriched region.

The overall number of SNPs was very similar among the six *P. aeruginosa* isolates in comparison to PAO1, demonstrating that most SNPs were commonly shared by all SPM-1-producing isolates (25%), except by PA11803, which exhibited approximately 400 more SNPs than the other five isolates (37%) (Table S9). The SNPs found in PA11803 were not homogeneously dispersed in the genome; instead, they were concentrated in a region located between 2,484,263 and 2,737,996 bp, a SNP hot spot measuring approximately 250 Kb in length (Figure 7). It is not uncommon to find these hot spot characteristics, as previously reported for other *P. aeruginosa* strains when using PAO1 as a reference genome (Bezuidt et al., 2013). Approximately 63% of SNPs in this region were classified as synonymous coding, followed by 22% classified as non-synonymous coding and 15% classified as intergenic. Most non-synonymous coding SNPs were found in genes predicted to encode hypothetical proteins (45%), but some were detected in the putative operon *ambB*-*E* and the *pvd* gene cluster (Table S10).

Among the SNPs shared by all *P. aeruginosa* isolates, the majority were located in the intergenic regions, followed by SNPs in CDSs, but without an amino acid change (synonymous coding). SNPs causing amino acid changes were also found in all six *P. aeruginosa* isolates (Table S9). We focused on this class of SNPs that could induce changes in phenotype. The remaining SNP classes were mostly found in hypothetical genes, except for a hydrolase, an amidase and an ABC transporter gene that harbored a lost stop codon (Table S10).

The SNP call performed using the PA1088 genome as a reference showed a number of SNPs lower than that detected in the previous analysis because of the high similarity among the isolates. Most of the non-synonymous coding SNPs found in genomic islands were located in genes predicted to encode hypothetical proteins, except for those encoding an ATP-dependent CLP protease found in PAGI-23 (PA3448), a zonula occludens toxin gene found in PAGI-29 (PA3448, PA7790, PA11803) and a lytic enzyme found in PAGI-32 (PA7790, PA8281) (Table S11).

### Microbiological characterization of bacterial isolates and multidrug-resistance mechanisms analysis

The six SPM-1-producing *P. aeruginosa* isolates were fully susceptible to polymyxin B (MICs, 0.25–0.5 μg/mL) but were resistant to ciprofloxacin (MICs, >32 μg/mL) and all β-lactams tested, except for aztreonam (Table 3). *P. aeruginosa* isolates exhibited intermediate susceptibility to this compound, which is not surprising because aztreonam is not recognized as a substrate by β-lactamases such as SPM-1, AmpC, OXA-50, and OXA-56 (Toleman et al., 2002; Lister et al., 2009; Leonard et al., 2013). All *P. aeruginosa* isolates were resistant to both amikacin and gentamicin, except PA3448. This isolate was susceptible to amikacin but resistant to gentamicin.

**Table 3:** **Microbiological characteristics of the six SPM-1-producing ***P. aeruginosa*** ST277 clinical isolates^**a**^**.

**ID isolate**	**Minimal inhibitory concentration (**μ**g/mL)**									
	**AMK**	**GEN**	**CAZ**	**CPM**	**ATM**	**PTZ**	**IMI**	**MER**	**CIP**	**PB**
PA1088	>128	>64	>32	>32	4	128/4	32	>64	>32	0.5
PA3448	8	32	>32	>32	8	128/4	>64	>64	>32	0.25
PA7790	>128	>64	>32	16	4	64/4	8	8	>32	0.5
PA8281	>128	>64	>32	>32	8	>128/4	>64	>64	>32	0.25
PA11803	>128	>64	>32	>32	16	>128/4	>64	>64	>32	0.25
PA12117	>128	>64	>32	>32	8	128/4	>64	>64	>32	0.5

a *Abbreviations: AMK, amikacin; ATM, aztreonam; CAZ, ceftazidime; CIP, ciprofloxacin; CPM, cefepime; GEN, gentamicin; ICU, intensive care unit; IMI, imipenem; MER, meropenem, PB, polymyxin B; PTZ, piperacillin/tazobactan*.

**Figure 8:**
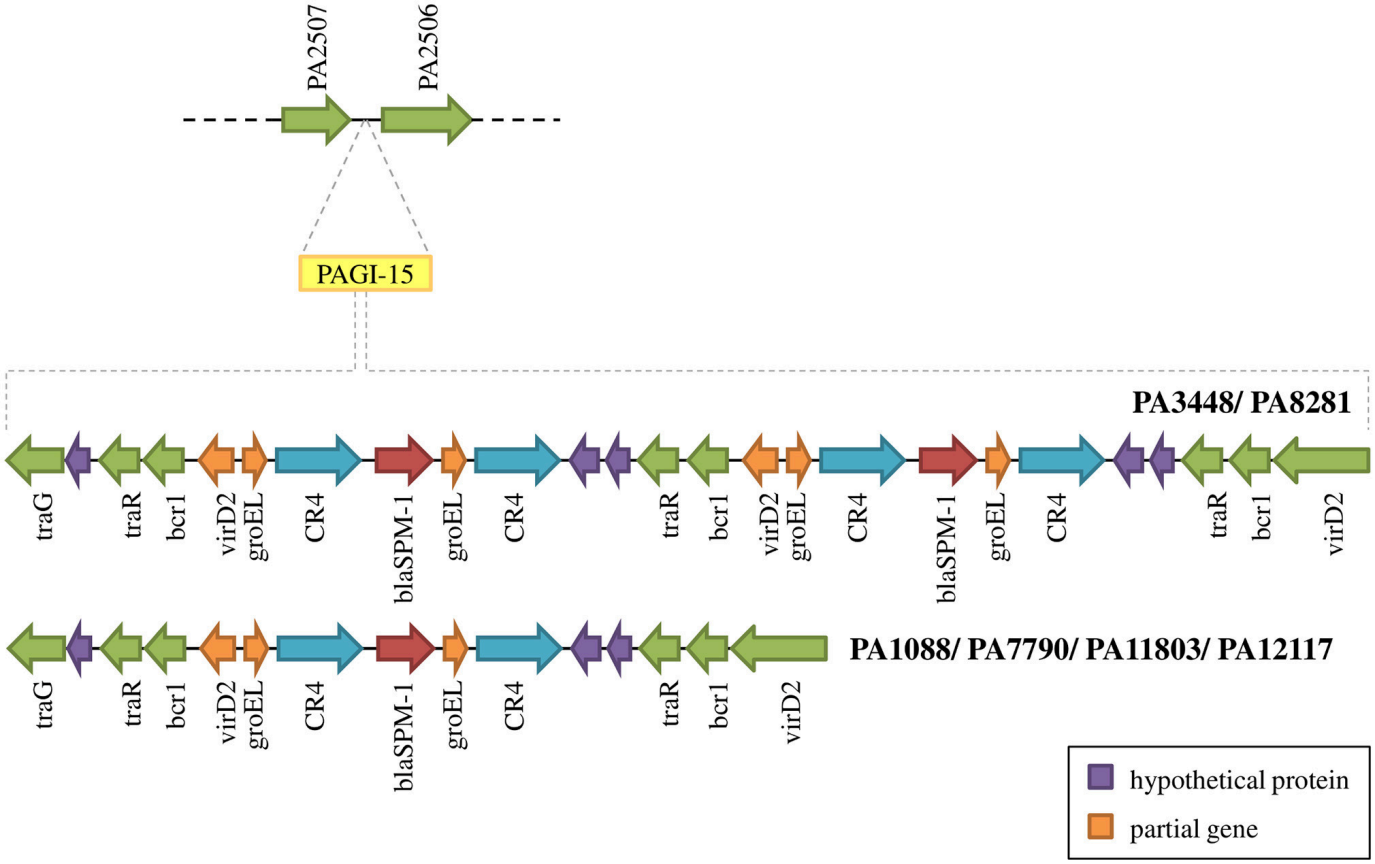
**Schematic overview of PAGI-15 region harboring the ***spm-1*** gene**. PA3448 and PA8281 carry two copies of this gene as a result of a duplication inside the island.

**Figure 9:**
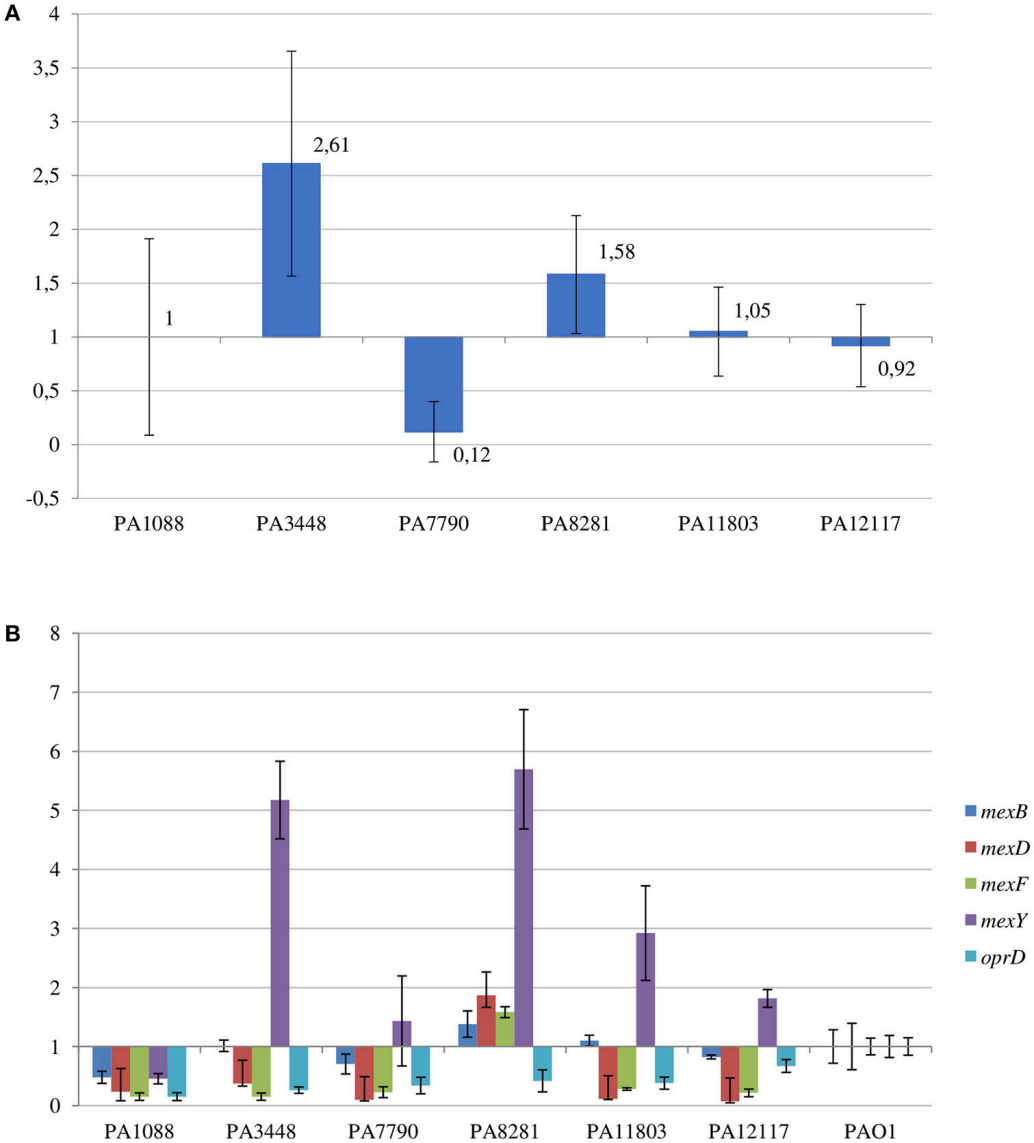
**Relative transcriptional levels by qRT-PCR of (A)** *bla*_SPM−1_ gene in the SPM-1-producing *P. aeruginosa* isolates compared to PA1088 strain; and **(B)** *mexB, mexD, mexF, mexY*, and *oprD* genes in the SPM-1-producing *P. aeruginosa* isolates compared to PAO1 strain.

The SPM-1 encoding gene, *bla*_SPM−1_, was found in all six *P. aeruginosa* isolates within two distinct genetic contexts (Figure 8). The *bla*_SPM−1_ gene was carried by a transposon with two CR4 elements in PAGI-15. The isolates PA1088, PA7790, PA11803, and PA12117 showed a duplication of a 4.2 Kb flanking sequence with two directly oriented copies of a region carrying one gene coding a hypothetical protein, one *traR*, one *bcr1*, and one *virD2* genes. This context is similar to that described for ICE_Tn4371_6061 (Fonseca et al., 2015), except that the transcription direction was inversely oriented since PAGI-15 is located in a genomic region that suffered a major chromosomal inversion. In the remaining two SPM-1-producing *P. aeruginosa*, PA3448, and PA8281, we observed a duplication of a region measuring approximately 10 Kb possibly caused by recombination of the directly oriented repeats aforementioned, between which *bla*_SPM−1_ was inserted, resulting in one additional copy of this gene (Wozniak and Waldor, 2010; Reams et al., 2012). The *bla*_SPM−1_ transcriptional levels were 2.6 and 1.6 times higher in the PA3448 and PA8281 isolates, respectively, compared with the transcription level of PA1088, suggesting that both *bla*_SPM−1_ copies were expressed (Figure 9A).

To verify any difference in genes involved in multidrug resistance mechanisms in all six isolates relative to the PAO1 reference strain, we performed BLASTp searches using default parameters. The chromosomal β-lactamases *bla*_OXA−50h_ and the cephalosporinase AmpC were detected among all six *P. aeruginosa* isolates. AmpC carried substitutions at R79Q and T105A that were identical to those described in the PDC-5 variant previously described (Rodríguez-Martínez et al., 2009). In addition, substitutions in AmpC regulators such as DacB (PBP4; A394P) and AmpD (G148A and S175L) were also observed in our study. However, no alterations in AmpD homologous proteins AmpDh2 and ApmDh3 were detected. Among the six *P. aeruginosa* isolates evaluated in this study, we also observed a substitution (A104P) in PbpC, a penicillin binding protein (PBP3A), which was detected by our SNP call analysis but only in the PA11803 isolate.

Other antimicrobial resistance genes were detected among *P. aeruginosa* isolates. The β-lactamase *bla*_OXA−56_, a narrow-spectrum oxacilinase; the aminoglycoside-modifying enzymes (AMEs) *aadA7* and *aac(6*′*)-Ib-cr*; the sulphonamide resistance gene *sul1*; and the chloramphenicol-related resistance gene *cmx* were present in three distinct mobile genetic contexts carried by a TnAs3 transposon, which were inserted into the PAGI-25 (Figure 6). *aac(6*′*)-Ib-cr, bla*_OXA−56_, and *aadA7* were harbored as gene cassettes of *In*163, a class 1 integron. These mobile genetic elements were integrated into the chromosome of all six *P. aeruginosa* isolates evaluated. We also observed two copies of *sul1* in all isolates, except in PA3448.

The six *P. aeruginosa* isolates showed a deletion in *oprD* (coding the main porin for the uptake of carbapenems), as outlined before, and an important reduction in the *oprD* transcriptional levels by qRT-PCR when compared to that of PAO1 strain (Figure 9B).

Substitutions at quinolone resistance determining regions of GyrA (T83I) and ParC (S87L) were observed, justifying the ciprofloxacin resistance exhibited by all *P. aeruginosa* isolates. In addition, an unknown substitution H262Q in ParC was observed.

Among the RND-type efflux systems present in *P. aeruginosa*, a Q25L substitution in the MexR was only observed in the PA1088 isolate suggesting that MexAB-OprM was not overexpressed in most isolates. In all six clones, the *mexZ* sequence was incomplete likely leading to the overexpression of MexXY-OprM system. In contrast, substitutions (F172I, I341F and D345E for MexT; D249N for MexS) not related to date to the MexEF-OprN system overexpression were observed in both transcriptional regulators, MexT and MexS, in all six isolates. By qRT-PCR, comparing the results to those obtained for PAO1 strain, increased *mexY* transcriptional levels were observed for PA8281 (5.7-fold), PA3448 (5.2-fold), PA11803 (2.9-fold), PA12117 (1.8-fold), and PA7790 (1.6-fold). On the other hand, a not significant increase in the *mexB* transcriptional levels was observed only for PA8281 (1.4-fold, Figure 9B).

### Virulence-related factors analysis

A BLASTn search using the Virulence Factors of Pathogenic Bacteria (VFDB) database (Chen et al., 2016) to find homology between sequences of the genomic islands found in SPM-1-producing *P. aeruginosa* isolates showed no significant results. Moreover, the comparative analysis did not yield any results related to genes encoding the main known virulence-related factors (Table S4).

The six SPM-1-producing *P. aeruginosa* showed several SNPs in genes encoding virulence factors (Table S12). We found 19, 20, and 19 non-synonymous SNPs in *cupC1, 2*, and *3*, respectively. These genes participate in the chaperone-usher pathway involved in biofilm formation (Vallet et al., 2004). Moreover, the S → Y SNP, previously reported in CupC2 (Bezuidt et al., 2013), was also present in our SNP call analysis only in PA3448. Our analysis also identified polymorphisms in genes related to virulence, such as *clpV3*, encoding a protease related to type VI secretion system (T6SS). We observed a non-synonymous SNP in *clpV3* that has not been related until now; however, it is known that mutations in this gene can cause inactivation of T6SS (Hachani et al., 2011). In addition, the PA11803 isolate has one ClpV3 SNP (I → T) that is different from another isolates (L → F), but it is also not clear whether this SNP could affect T6SS. The vgrG1 gene is also related to T6SS, and it is responsible for encoding a protein that acts as a puncturing device (Hachani et al., 2014). Four isolates (PA3448, PA8281, PA11803, and PA12117) showed a non-synonymous coding SNP (N → D) that was previously identified (Bezuidt et al., 2013). The *pvd* operon plays a role in the pyoverdine pathway and is related to iron acquisition. Mutations in this operon could decrease the virulence of *P. aeruginosa* (Lehoux et al., 2000). Our analysis revealed polymorphisms in pvdA, pvdQ, pvdR, and pvdT, but only pvdR and pvdT showed non-synonymous SNPs, in contrast to a previous report (Bezuidt et al., 2013). The ptxR gene is another gene related to the virulence process and is involved in quorum sensing in *P. aeruginosa* (Carty et al., 2006). We found one non-synonymous coding SNP in this gene in PA11803 (S → G). The pilY1 gene is involved in pilus assembly, twitching motility and adhesion to host cells. All six isolates showed a non-synonymous SNP (F → Y) in PilY1 that was previously described (Bezuidt et al., 2013).

We found SNPs in the *amb* operon, which produces l-2-amino-4-methoxy-trans-3-butenoic acid (AMB), a potent antibiotic and toxin (Lee et al., 2010). PA11803 showed one non-synonymous SNP in *ambB* and *ambD*, three in *ambC* and eight in the *ambE*. It has been shown that some site mutations abolish AMB production (Lee et al., 2010), but in our case, more studies are necessary to elucidate whether these SNPs could cease the toxin production.

We identified polymorphisms among the six isolates located in genomic islands when using PA1088 as a reference genome. Our analysis identified a non-synonymous coding SNP (V → D) in a gene encoding a protein homologous to zonula occludens in PA3448, PA7790 and PA11803. The zonula occludens is an enterotoxin elaborated by Vibrio cholerae that increases intestinal permeability by interacting with a mammalian cell receptor with subsequent activation of intracellular signaling leading to the disassembly of intercellular tight junctions (Di Pierro et al., 2001).

## Discussion

*P. aeruginosa* is an important pathogen in the nosocomial environment (Buhl et al., 2015). In this study, we characterized the full genome of six SPM-1-producing *P. aeruginosa* ST277 isolated from a Brazilian teaching hospital. This clone is widely spread within Brazilian hospitals and, although less frequently distributed than ST111 and ST235, has been recognized as a multidrug-resistant *P. aeruginosa* global clone (Kos et al., 2015; Oliver et al., 2015; van Belkum et al., 2015).

Most of the genomic features described are consistent with those reported for previously sequenced genomes of other *Pseudomonas* (Silby et al., 2011). The genomes had a size between 6.6 and 7 Mb with a minor variation in chromosome size and CDS numbers caused mainly by the acquisition of genomic islands. Despite these insertions, the overall functional classification of the CDSs was quite similar when comparing our six isolates with PAO1, except for additional CDSs grouped in the replication and repair category (Table S2). All isolates were observed in three additional CDSs predicted to encode an SSB protein. These proteins protect single-stranded DNA from degradation and are reported to play a role in the mobilization of other proteins in the process of DNA replication, recombination or repair (Shereda et al., 2008). The ABC excinuclease subunits A and B, involved in the recognition and removal of damaged DNA, were also present in all six isolates (Verhoeven et al., 2002). The presence of these replication- and repair-related proteins suggests a reinforcement of mechanisms for maintaining DNA integrity. In addition to these mechanisms, we identified a type I-C CRISPR-Cas system in PAGI-34. The CRISPR-Cas genes constitute a bacterial adaptive genetic immune system that plays a major role in controlling horizontal transfer of elements such as phages and plasmids, avoiding the insertion of mobile elements that could cause gene or operons interruptions or genetic rearrangements. The observation of the type I-C CRISPR-Cas systems in multidrug-resistant *P. aeruginosa* group ST277 carried by an acquired mobile element is a rare event and was recently described (van Belkum et al., 2015). Moreover, the presence of a type I-C CRISPR-Cas system has been correlated with resistance to amikacin and the presence of the *rmtD, aad7* and *bla*_*OXA*_-encoding resistance genes (van Belkum et al., 2015). Thus, the CRISPR-Cas system could be in part responsible for the genomic stability of the six *P. aeruginosa* isolates. Based on our phylogenetic analysis, GI and IS detection (Figures 2, 5, 6, respectively), we hypothesized that this system would have been recently interrupted in the PA7790 and PA8281 isolates, and lost in PA11803 and PA12117 isolates. However, prior to these events, the type I-C CRISPR-Cas system could have been fully functional, playing its role in the genomic plasticity of these isolates in past years. This hypothesis and the protective effect of the type I-C CRISPR-Cas system need to be further investigated.

The presence of IS elements is related to horizontal gene transfer and genomic rearrangement (Kung et al., 2010; Al-Nayyef et al., 2015). The overall number of ISs in the SPM-1 isolates was quite similar. Slight differences can be observed between them according to the rearrangement or presence of specific genomic islands when the genomes are compared.

The SPM-1-producing *P. aeruginosa* clones evaluated in this study were fully resistant to all β-lactams, including carbapenems. The production of metallo-β-lactamases such as SPM-1 has been recognized as the main mechanism of carbapenem resistance among *P. aeruginosa* (Toleman et al., 2002; Lister et al., 2009; Kos et al., 2015). In addition, other mechanisms of β-lactam resistance were identified in these isolates, including loss of OprD, production of intrinsic and acquired β-lactamases, and overexpression of efflux systems. OprD serves as the preferred portal of entry for the carbapenems into the bacterial cell, and the loss of OprD significantly decreases the susceptibility of *P. aeruginosa* to available carbapenems, especially imipenem (Lister et al., 2009). OprD loss has been commonly reported as a mechanism of resistance to carbapenems, in *P. aeruginosa* isolated from Brazilian medical centers (Xavier et al., 2010; Fehlberg et al., 2012; Ocampo-Sosa et al., 2012; Cavalcanti et al., 2015; Kos et al., 2015).

Genes encoding intrinsic β-lactamases such as AmpC and OXA-50 h were also encountered in the evaluated genomes. Wild-type strains of *P. aeruginosa* produce only low basal levels of AmpC and are susceptible to antipseudomonal penicillins, penicillin-inhibitor combinations, cephalosporins, and carbapenems. When AmpC production is significantly increased, *P. aeruginosa* develops resistance to all β-lactams, with the exception of the carbapenems (Lister et al., 2009). However, it has been demonstrated that some AmpC variants are also able to hydrolyse carbapenems. The *ampC* carried by all SPM-1-producing *P. aeruginosa* isolates was identical to the PDC-5 variant and might have contributed to a decrease in susceptibility to oxyiminocephalosporins and imipenem (Rodríguez-Martínez et al., 2009). In addition, AmpC could be overexpressed by the ST277 clone because mutations were observed in *ampD*, a negative regulator of AmpC, and *dacB*, which codifies a non-essential low-molecular-weight PBP4 gene that was previously identified as an important component of *ampC* regulation (Moya et al., 2009). The *bla*_SPM−1_ gene was located in PAGI-15, and it was duplicated in two SPM-1 producers. PAGI-15 without the *bla*_SPM−1_ duplication can be found in at least five different *P. aeruginosa* ST277 strains previously sequenced (CCBH4851, PS106, 9BR, 19BR, and 213BR). The island without any copy of *bla*_SPM−1_ appears to be widely spread among other bacteria, including *Pseudomonas* and other genera, such as *Ralstonia oxalatica*. Together, these data suggest the recent insertion of this transposon and the acquisition of *bla*_SPM−1_ gene by *P. aeruginosa*.

Mechanisms of aminoglycoside resistance, such as the production of AMEs and rRNA methylases, were detected among the SPM-1-producing *P. aeruginosa* isolates (Doi et al., 2007a). The *aadA7* and *aac(6*′*)-Ib-cr* genes were found in the six *P. aeruginosa* isolates. Whereas the *aadA7* gene codifies an AME capable of modifying the molecular structure of streptomycin and spectinomycin by adenylation, *aac(6*′*)-Ib-cr* codifies an acetyltransferase, AAC(6′)-Ib-cr, that acetylates not only the molecular structure of kanamycin, tobramycin and amikacin but also that of ciprofloxacin (Robicsek et al., 2006; Ramirez and Tolmasky, 2010). In addition, three *P. aeruginosa* isolates also carried *rmtD*, a gene encoding RmtD, a rRNA methylase. The methylation of the 16S rRNA of the A site of the 30S ribosomal subunit interferes with aminoglycoside binding and promotes high-level resistance to all clinically available aminoglycosides (Doi and Arakawa, 2007). *P. aeruginosa* co-producing RmtD and SPM-1 have been frequently reported among Brazilian isolates (Doi et al., 2007b; Lincopan et al., 2010). At least three distinct mechanisms could be related to fluoroquinolone resistance profile observed among the SPM-1-producing *P. aeruginosa* isolates: the known *gyrA* and *parC* mutations, the presence of *aac(6*′*)-Ib-cr*, and possible overexpression of MexXY-OprM efflux systems.

Treatment of infections caused by SPM-1-producing *P. aeruginosa* is currently problematic because only polymyxins remain active. The combination of old antibiotics such as chloramphenicol or bicyclomycin may not be a valid strategy for the treatment of such infections because genes encoding mechanisms of resistance to chloramphenicol (*cmx-1*) and bicyclomycin (*bcr1*) were presented in the genome of all sequenced *P. aeruginosa* isolates.

The pathogenicity of *P. aeruginosa* has been attributed to the production of several virulence factors, among them are pili, exotoxins, pyoverdin, secretion systems, biofilm formation, all of which tightly controlled by regulatory systems (Balasubramanian et al., 2013; Gellatly and Hancock, 2013). Although we found alterations in several genes encoding virulence factors such as: *clpV3*, gene components of the *pvd* cluster, *cupC* and *pilY1*, the impact of these findings on their transcription has not yet been studied and need to be further investigated. Surprisingly, the *clpV3* SNP found in our analysis has never been described. We also found several possible new transcriptional regulators carried by the acquired genomic islands, such as genes sharing a homology with *prtN, ptrB* and *prtR*, which appears to have their expression affected when exposed to β-lactam stress (Matsui et al., 1993; Balasubramanian et al., 2012). These finding suggest additional players in *P. aeruginosa* regulatory network and, consequently, in the bacterial response to the antibiotic therapy.

Despite the slight variations observed among the SPM-1-producing *P. aeruginosa* isolates, our work demonstrated, by comparative genomics, IS and SNP analysis, that the six isolates did not present high genome plasticity over the 9-year period even after being exposed to an environment of antibiotic selective pressure. We attributed this finding to the presence of additional replication- and repair-related proteins and the type I-C CRISPR-Cas system in PAGI-34 because these factors could be responsible for modulating the shape of the *P. aeruginosa* genome. This comparative genomics report is an important way of determining strain features to enable the development of new therapies to combat infections and to avoid the occurrence of future outbreaks and the worldwide dissemination of the SPM-1-producing *P. aeruginosa* ST277 strains, which despite being widely spread only in Brazilian hospitals, have already been recognized as multidrug-resistant global clones.

## Author contributions

AN performed genome annotation, comparative genomics, interpreted the results and wrote the manuscript. MO performed insertion sequences and phylogeny, analysis and wrote the manuscript. WM performed microbiological characterization. GM performed the single-nucleotide polymorphisms analysis and wrote the manuscript. LF performed microbiological characterization. LA performed the genome assembly. LC performed genome analysis and revised the manuscript. AG and AV designed the experiment, supervised the research, revised the manuscript and served as corresponding authors.

## Funding

This work was funded by National Counsel of Technological and Scientific Development (CNPq) (grant numbers: 305535/2014-5, 302768/2011-4, 312864/2015-9), Fundação de Amparo à Pesquisa do Estado do Rio de Janeiro (FAPERJ) (grant number: E-26/202.903/2016) and Coordenação de Aperfeiçoamento de Pessoal de Nível Superior (CAPES).

### Conflict of interest statement

The authors declare that the research was conducted in the absence of any commercial or financial relationships that could be construed as a potential conflict of interest.

## Footnotes

^1^ANVISA. “Relatório de resistência microbiana em infecções primárias de corrente sanguínea confirmadas laboratorialmente associadas a cateter venoso central em unidades de terapia intensiva (2014)”. Last modified December 31, 2015. http://www20.anvisa.gov.br/segurancadopaciente/index.php/publicacoes/item/12.

^2^PubMLST. “*Pseudomonas aeruginosa* MLST”. Accessed May 15, 2015. http://pubmlst.org/paeruginosa.

^3^Babraham Bioinformatics. “FastQC”. Last modified March 08, 2016. http://www.bioinformatics.babraham.ac.uk/projects/fastqc.

^4^FASTX-Toolkit. “FASTQ/A short-reads pre-processing tools”. Last modified February 02, 2010. http://hannonlab.cshl.edu/fastx_toolkit/index.html.

^5^Picard Tools by Broad Institute. “Picard”. Accessed December 02, 2015. http://broadinstitute.github.io/picard.

^6^EUCAST. “Clinical breakpoints”. Accessed January 18, 2016. http://www.eucast.org/clinical_breakpoints.
