# Supplementary figures and images for: Intraclonal Genome Stability of the Metallo-β-lactamase SPM-1-producing Pseudomonas aeruginosa ST277, an Endemic Clone Disseminated in Brazilian Hospitals
Source: Front Microbiol. 2016 Dec 5;7:1946. doi: 10.3389/fmicb.2016.01946 (PMC5136561; doi:10.3389/fmicb.2016.01946)

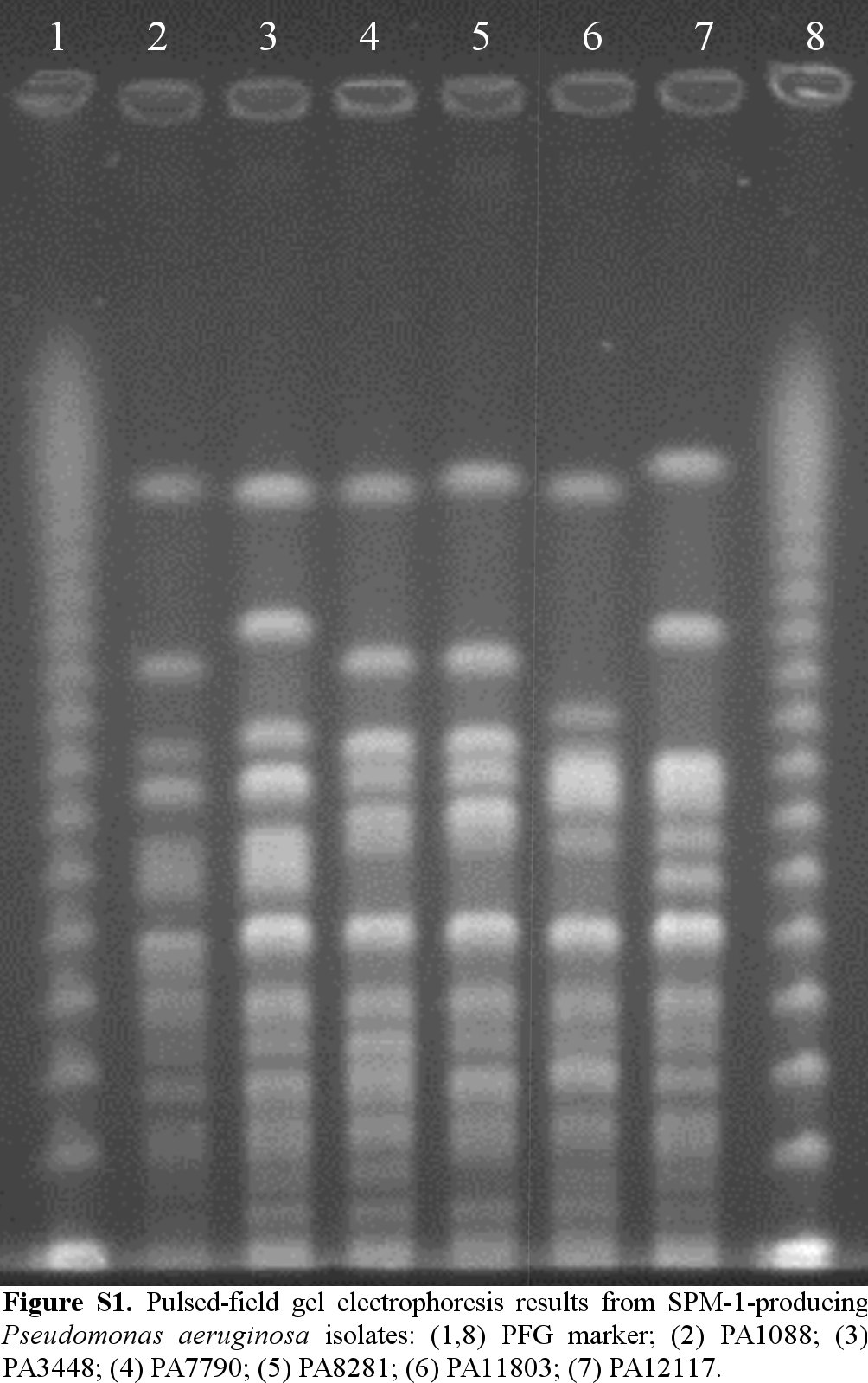

Supplement: Supplementary file 13 [file Image1.TIF]

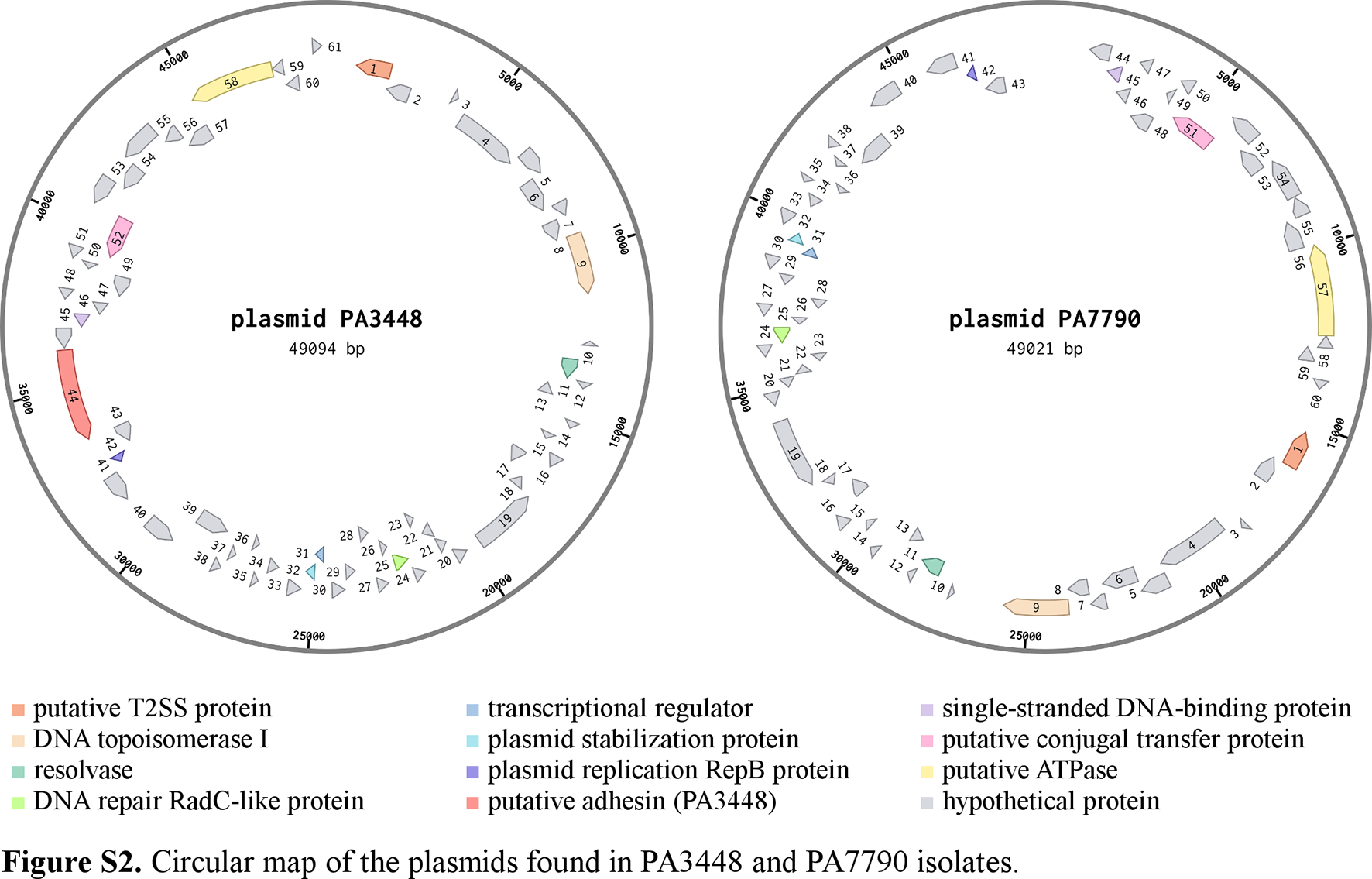

Supplement: Supplementary file 14 [file Image2.TIF]

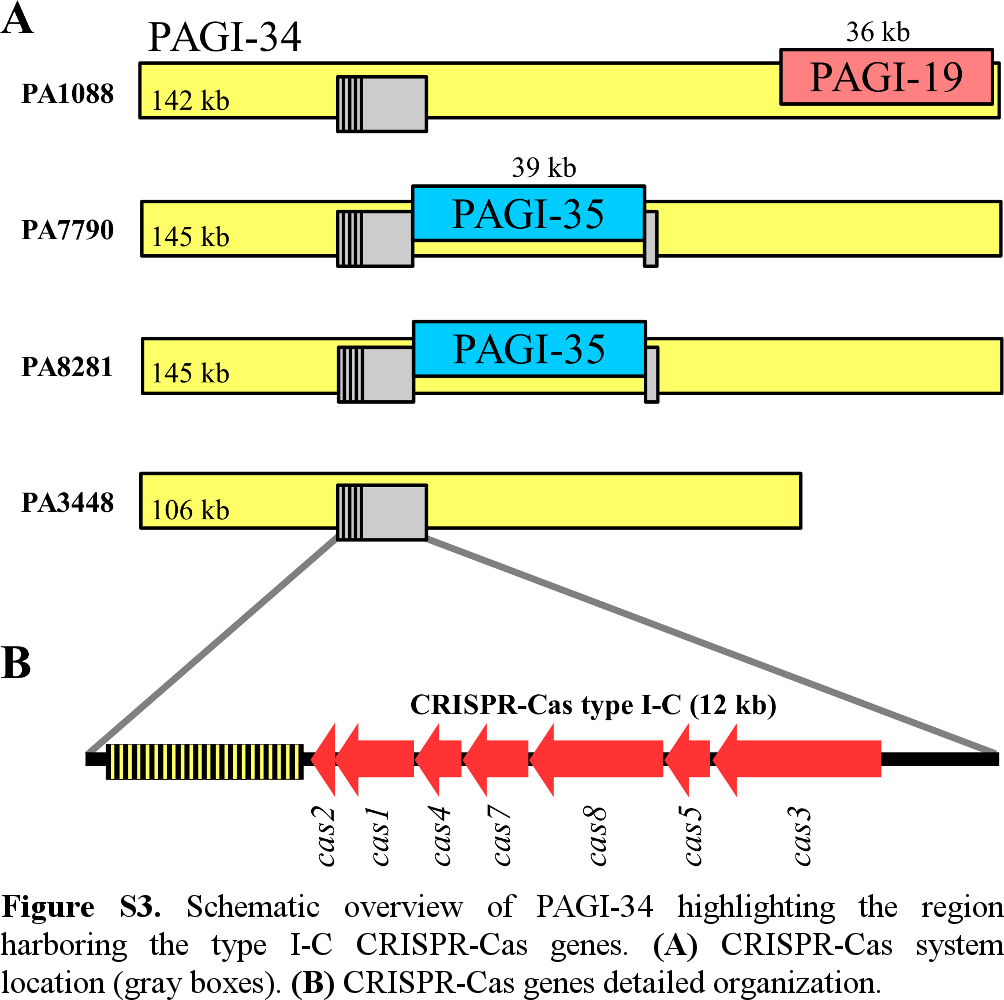

Supplement: Supplementary file 15 [file Image3.TIF]
